# The Epistemic Value of Gain of Function Experiments

**DOI:** 10.1128/msphere.00714-23

**Published:** 2023-12-22

**Authors:** Arturo Casadevall, Ferric C. Fang, Michael J. Imperiale

**Affiliations:** 1 Department of Molecular Microbiology and Immunology, Johns Hopkins Bloomberg School of Public Health, Baltimore, Maryland, USA; 2 Departments of Laboratory Medicine and Microbiology, University of Washington, Seattle, Washington, USA; 3 Department of Microbiology and Immunology, University of Michigan, Ann Arbor, Michigan, USA; The University of Arizona, Tucson, Arizona, USA

**Keywords:** biosafety, biosecurity, viruses

## Abstract

The phrase “gain of function” (GOF) has recently acquired a negative connotation in experimental biology by its association with risky science. Whereas much of the discussion on the relative merits of GOF-type experiments has focused on their risk-benefit equation, relatively little has been said about their epistemic value. In this article, we recount how GOF experiments were critical for establishing DNA as the genetic material, the identification of cellular receptors, and the role of oncogenes in cancer research. Today, many of the products of the biomedical revolution such as synthetic insulin, growth factors, and monoclonal antibodies are the result of GOF experiments where cells were given the new function of synthesizing medically important products. GOF experiments and complementary loss of function experiments are epistemically powerful tools for establishing causality in biology.

## PERSPECTIVE

Federal and state efforts have been recently introduced to legislate against or otherwise regulate “gain of function” research, defined as any research that could “enhance the transmissibility, virulence, or pathogenicity of any pathogen or non-pathogen agent in a way that could increase the agent’s transmissibility, virulence, or pathogenicity in humans” (1). In fact, the U.S. House of Representatives passed an amendment on 14 November 2023, prohibiting the use of federal funds to support “gain of function” research (2). The phrase “gain of function” (GOF) is a long-standing term in the biological sciences that refers to an experimental manipulation in which an organism, cell, or microbe acquires a new function. Its use in microbiology research first broke into the public consciousness in 2012, when there was extensive media coverage of two manuscripts reporting experiments that modified the H5N1 avian influenza virus so that it could be transmitted between mammals (3). The original meaning of the phrase GOF at that time largely disappeared from use except in the science policy world, where efforts were made to develop mechanisms to oversee GOF experiments with especially dangerous pathogens, i.e., those with the ability to cause a pandemic. Instead, the terms “gain of function research of concern” and “enhanced pathogens with pandemic potential” were introduced to describe this small and specific subset of GOF research, and the U.S. government developed a framework for dealing with such work (4). Nevertheless, the GOF designation, along with the phrase “loss of function” (LOF), has remained in scientific use with regard to mutations that affect carcinogenic potential (5), neuronal function (6), and more. Following the onset of the COVID-19 pandemic, GOF research reemerged into the public discourse after the unsubstantiated speculation that SARS-CoV-2 might have been genetically engineered at the Wuhan Institute of Virology in China (7). Unfortunately, the distinction between GOF research with potential risks to public health and the vast majority of GOF research, which presents little or no such risk, has become increasingly blurred. Here, we would like to discuss what GOF is and why it is an essential tool for microbiologists.

As we know, the diversity of life is a product of evolution, and the raw material of evolutionary change is the information encoded in nucleic acid sequences. A function is a “phenotype,” an observable property of an organism, whereas the genetic code underlying a specific function is a “genotype.” When Gregor Mendel attempted to determine the principles of inheritance in pea plants, he recorded the phenotypes in successive generations of plants and inferred that there must be underlying genotypic changes that were responsible, although at the time, the nature of genes was not known and would not be known for another century. Mutations are constantly being generated in all living organisms, mostly arising from base substitutions, insertions, deletions, recombination, and horizontal gene transfer; in segmented viruses and sexual organisms, variation is also created by gene reassortment. The correlation between genotype and phenotype is complex and often unpredictable and must be confirmed experimentally. Scientists may observe the effects of natural genotypic variation, enhance the rate of mutation by treatment with a mutagen, or construct targeted genetic changes using molecular biology methods and determine the functional consequences of these changes. Whether a particular function, such as binding to a receptor, resistance to an antibiotic, or virulence in a specific host, is gained or lost can only be determined by testing the phenotype, and in some cases, procedures to select or enrich for a specific function may be performed. Notably, any new function is only recognized if it is measured and/or selected for, so most phenotypes that are gained or lost through genetic changes are unrecognized. Richard Lenski demonstrated this in classical studies of experimental evolution in which he cultured *Escherichia coli* under different conditions and identified novel mutants with enhanced fitness in the presence of specific sugars (8). Lenski did not endow the bacteria with new qualities; he simply grew them under specific conditions and then identified a subpopulation with a GOF that allowed them to thrive under these conditions. Indeed, this is a basic principle of mutations, as elegantly demonstrated by Luria and Delbruck 80 years ago (9). Such experiments are constantly occurring everywhere in the natural world, where most genetic variation and phenotypic selection take place. Such experimental studies of genotypic variation in laboratories are merely mimicking these natural processes. Of the nearly 1,500 known microbes that are pathogenic for humans, all have been created by nature.

GOF research in which specific microbes acquire new functions is at the heart of microbiology and has been so nearly since the inception of the field in the 19th century. Before rabies was identified as a virus, in fact before viruses were even known to exist, Louis Pasteur and Émile Roux were passaging brain tissue from rabid animals into other animals. They discovered that serial passage of rabies-infected tissue in dogs shortened the incubation period of the disease, suggesting a GOF resulting in increased virulence. Eventually, they determined that desiccating neural tissue from rabbits infected with the virus seemed to attenuate the virulence, and this material could be inoculated into dogs to make them immune to rabies (10). Conceptually, the experiments of Pasteur and Roux are not fundamentally different from those performed by the Kawaoka and Fouchier laboratories when they engendered controversy by passaging the H5N1 influenza virus in ferrets to see how the virus adapted (11, 12). The difference, rather, is in the potential risks due to the transmission properties of rabies virus vs. influenza virus. The attenuated bacille Calmette-Guerrin (BCG) strain of *Mycobacterium bovis* that has been safely administered to billions of people to stimulate immunity to tuberculosis was generated by 230 serial passages at the Institut Pasteur between 1908 and 1919 (13). Although some may balk at grouping virulence attenuation with gain of function, this example shows how GOF and LOF can be a matter of perspective. In fact, the original selection that generated the BCG strain consisted of *in vitro* growth on potato medium supplemented with glycerin and ox bile. Selection of variants with an increased ability to grow on this medium would be anticipated, and in fact, restoration of one of the genes found to be deleted in BCG results in slower *in vitro* growth of the parental BCG strain (14). Hence, the BCG strain gained the function of rapid growth in the new medium, and this correlated with attenuated virulence, but the latter could only be demonstrated empirically. When used for vaccination, the BCG strain acquired the new function of infection without eliciting disease, which induced a protective immune response while manifesting LOF in virulence. The BCG example illustrates the need for nuance when considering both GOF and LOF.

Alternatively, new functions may have nothing to do with virulence and can be utilitarian and non-controversial, such as having bacteria or yeasts express the hormone insulin that is used to treat diabetes. A partial list of useful GOF experiments ranging from enhanced batteries to cancer immunotherapy is provided in reference (15). Some utilitarian GOF experiments such as genetically modifying plants to increase yield or pest resistance, commonly referred to as “genetically modified organisms,” remain controversial, and their use in agriculture is banned in some countries. GOF experimentation has been controversial because of safety or ethical concerns, but the epistemic value of introducing or identifying new functions to learn their purpose is rarely discussed. GOF experiments have played a critically important role in the development of molecular biology and remain a powerful tool for establishing causality and certainty in the biological sciences. Following are just a few examples:

### The molecular biology revolution

GOF experiments have a storied place in the molecular biology revolution. In 1928, Frederick Griffith reported that when he mixed heat-killed virulent pneumococcal bacteria with live avirulent bacteria and inoculated a mouse, he could recover virulent bacteria (16). He correctly deduced the existence of what he called a “transforming principle.” Although he could not have known it at the time, he was witnessing the transfer of DNA from dead virulent bacteria into avirulent bacteria, which allowed the latter to survive in the mouse. He had conducted a GOF experiment in which avirulent bacteria acquired the capacity for virulence. Later, Oswald Avery and his colleagues would show that the transforming principle was DNA, by performing a GOF experiment in which avirulent non-encapsulated pneumococci reacquired virulence from extracts of heat-killed virulent bacteria, which was abrogated by DNase treatment of the extracts (17). The Avery experiment was central in establishing that DNA carries the genetic code and set the stage for the molecular biology revolution.

### Receptor identification

Introducing the genes encoding a cellular receptor into a cell that does not naturally express that receptor is a powerful way to establish the specificity of ligand-receptor interactions. For example, inserting the genes coding for the IgG Fc receptor into cells that do not naturally express this receptor confers the transformed cells with the new function of antibody-mediated phagocytosis (18). This type of experiment unambiguously associates a gene in question with its function by giving cells a new ability. Furthermore, such experiments also validate the role of a ligand in triggering receptor activation.

### Cancer research

GOF experiments were essential in establishing the function of cellular oncogenes in the malignant transformation of normal cells into cancer cells (19). Oncogenes were first identified as sequences in retroviruses that caused tumors in animals. These viral sequences were subsequently found to be derived from host genes involved in the regulation of cell growth that had been co-opted by the virus. This was demonstrated functionally by introducing genomic DNA from cancer cells into normal cells and showing they became tumorigenic, followed by similar experiments in which a single gene cloned from the cancer cell genome could deliver the same phenotype, thus establishing its role in carcinogenesis.

### Transmissibility of avian flu viruses in mammals

While the risks of experiments to determine the transmissibility of highly pathogenic H5N1 avian influenza virus in mammals (11, 12) continue to be debated, it is often forgotten that these experiments provided new information by unequivocally establishing that this virus has the genetic potential for mammalian transmissibility and could thus launch a devastating human pandemic. Transmissibility is a complex trait that cannot be predicted from genotypic or tissue culture studies and must be established experimentally or epidemiologically. However, for microbes that have no prior history of transmission in humans, epidemiological methods cannot be used. The only way to establish with certainty whether a microbial species has the potential for mammalian transmissibility is to address the question experimentally. A GOF experiment allowing a microbe to acquire the function of transmissibility provides strong evidence that it possesses the biological potential for this trait to emerge naturally. This increases the urgency of developing measures to prepare for and prevent an H5N1 influenza pandemic and identifies some of the possible genetic and functional changes that might herald such a pandemic.

### Loss and gain of function in virulence studies

As the field of microbial pathogenesis embraced molecular biology in the 1980s, a new standard arose for establishing a causal link between a gene and virulence. Whereas a loss of function associated with a mutation was highly suggestive of causation, such associations were not definitive because other undetected mutations might still be contributing to the phenotype. Although one could increase certainty by showing that the same phenotype arose in multiple independent mutants, this approach still relied on inductive reasoning and did not provide conclusive evidence of causality. In the late 1980s, the bacteriologist Stanley Falkow proposed the use of gene complementation as the definitive method to establish the role of a specific gene or genes in virulence (20). According to this approach, the most rigorous method to associate a gene with virulence is to generate a mutant strain lacking the gene, demonstrate a loss of virulence, and then restore virulence by reintroducing a wild-type copy of the gene or genes. This approach, referred to as “molecular Koch’s postulates,” is considered to be superior in establishing a causal relationship in virulence because it doesn’t depend on probabilistic arguments from comparisons of independent mutants.

Nevertheless, there remains the problem of defining “virulence,” a complex property that is highly dependent on the host as well as on the microbe (21) and whose evolutionary dynamics are highly context dependent (22). Virulence requires a microbe to establish colonization of a host, replicate within the host, produce disease, and then perhaps spread to other hosts. This requires many independent functions, and genotypic changes may enhance one function while diminishing others, with unpredictable effects on overall virulence. Moreover, a microbe with increased virulence in one host may have reduced virulence in another. When Pasteur passaged rabies-infected tissue in different mammalian hosts, he could have selected for a more virulent virus, but fortunately, he found the opposite. Even LOF may increase the virulence of a microbe (23). Prohibiting all experimentation that could “enhance the transmissibility, virulence, or pathogenicity of any pathogen or non-pathogen agent in a way that could increase the agent’s transmissibility, virulence, or pathogenicity in humans” is intrinsically problematic because virulence cannot be predicted *a priori* and can only be determined experimentally.

### LOF, GOF, and the quest for causality

In the microscopic world, whether dealing with eukaryotic cell biology or microbiology, causes and effects cannot usually be observed directly and must be established through experimentation that employs indirect approaches, such as mutational analysis. Hence, the quest for causality usually requires a combination of experiments that are then linked by mechanistic associations. LOF and GOF experiments are approaches to establish causality, which in combination are epistemically complementary and, in some situations, synergistic, as neither LOF nor GOF experiments alone have the same epistemic power in isolation as they do in combination. Through genetic association studies, LOF can strongly suggest function but cannot definitively establish causality because the possibility of confounding by a hidden variable remains; ruling out all such potential hidden variables is akin to proving a negative connotation, i.e., a logical impossibility. In contrast, GOF experiments can unambiguously establish causality between the addition of a gene and a new property but cannot exclude the possibility that the new function requires the specific biological context created by the experiment. Moreover, GOF experiments are susceptible to the phenomenon of emergence, in which a new unanticipated property arises from introduction of a new variable into a complex system. In this regard, consider the controversial insertion of the IL-4 gene into the murine ectromelia virus (24), which was intended to improve immune responses to the virus but instead made the virus more lethal, providing a potential strategy to overcome vaccine immunity against human poxviruses (25). The complementary nature of LOF and GOF mutations in providing insights into function is illustrated by studies of the GABA receptor, in which LOF and GOF mutations produced different clinical outcomes (6). When LOF or GOF interventions abolish or confer a particular phenotype, respectively, they provide powerful evidence for causality. Further experiments, in particular mechanistic studies (26), can provide additional certainty. In the Fc receptor example mentioned earlier, LOF/GOF experiments implicated the receptor in antibody-mediated phagocytosis, but showing the specific role of antibodies required evidence of their direct interaction with the receptor. Others have also noted the close relationship between LOF/GOF experiments including that these share the same methodology and both are often done by the same research group (27).

### GOF as an epistemic tool

As is evident from the above examples, GOF experiments were critical in the molecular biology revolution and remain powerful tools to probe important biological questions. For some biological questions, such as the complex properties of microbial transmissibility and virulence, GOF experiments may provide the only tools available to provide answers with certainty. GOF experiments are essential not only for understanding how microbes cause disease but also to determine which parts of a microbe might be a suitable drug or vaccine target, how antimicrobial and anticancer drugs work, and how microbes become resistant to antimicrobial agents. The epistemic value of GOF experiments must therefore be considered alongside the easily translatable potential benefits and any biosafety and biosecurity risks. However, just because an experiment can be done doesn’t mean that it should be done. Rather than implementing blanket prohibitions or moratoriums on certain types of experiments, it is incumbent upon scientists and others to carefully consider the question being studied and its importance and then perform a risk-benefit calculation to decide whether the experiment is worth doing (28, 29). For some important questions, the risk of ignorance may outweigh the risks of performing the experiment. Of course, appropriate biosafety and biosecurity precautions must be taken and procedures assiduously followed.

The vast majority of GOF/LOF experiments pose no risk to humanity, and most risky experimentation is not GOF/LOF research. Highly pathogenic organisms pose a risk without needing to acquire new functions and are already subject to extensive oversight. Focusing on the concept of GOF rather than risk *per se* could impede research progress without improving safety and security. If all GOF research were to be prohibited or crippled by restrictive legislation, the consequences could be catastrophic. Humanity will be less safe if we are deprived of the essential knowledge that only GOF experiments can provide.
